# Artificial Intelligence (AI) Chatbots in Medicine: A Supplement, Not a Substitute

**DOI:** 10.7759/cureus.40922

**Published:** 2023-06-25

**Authors:** Ibraheem Altamimi, Abdullah Altamimi, Abdullah S Alhumimidi, Abdulaziz Altamimi, Mohamad-Hani Temsah

**Affiliations:** 1 College of Medicine, King Saud University, Riyadh, SAU; 2 Pediatric Emergency, Toxicology, King Fahad Medical City, Riyadh, SAU; 3 College of Medicine, King Saud Bin Abdulaziz University for Health Sciences, Riyadh, SAU; 4 Pediatric Intensive Care Unit, King Saud University, Riyadh, SAU

**Keywords:** human-machine collaboration, ai limitations, healthcare efficiency, regulatory measures, ethics in ai, patient care, medical professionals, healthcare, ai chatbots, artificial intelligence (ai)

## Abstract

This editorial discusses the role of artificial intelligence (AI) chatbots in the healthcare sector, emphasizing their potential as supplements rather than substitutes for medical professionals. While AI chatbots have demonstrated significant potential in managing routine tasks, processing vast amounts of data, and aiding in patient education, they still lack the empathy, intuition, and experience intrinsic to human healthcare providers. Furthermore, the deployment of AI in medicine brings forth ethical and legal considerations that require robust regulatory measures. As we move towards the future, the editorial underscores the importance of a collaborative model, wherein AI chatbots and medical professionals work together to optimize patient outcomes. Despite the potential for AI advancements, the likelihood of chatbots completely replacing medical professionals remains low, as the complexity of healthcare necessitates human involvement. The ultimate aim should be to use technology like AI chatbots to enhance patient care and outcomes, not to replace the irreplaceable human elements of healthcare.

## Editorial

The intersection of artificial intelligence (AI) and healthcare has been a hotbed for innovative exploration. One area of particular interest is the use of AI chatbots, which have demonstrated promising potential as health advisors, initial triage tools, and mental health companions [1]. However, the future of these AI chatbots in relation to medical professionals is a topic that elicits diverse opinions and predictions [2-3]. The paper, "Will AI Chatbots Replace Medical Professionals in the Future?" delves into this discourse, challenging us to consider the balance between the advancements in AI and the irreplaceable human aspects of medical care [2].

The rise of AI chatbots in healthcare

AI chatbots are playing an increasingly transformative role in the delivery of healthcare services. Their ability to process vast amounts of data and detect patterns and trends far beyond human capability makes them ideal for managing routine tasks such as scheduling appointments, sending medication reminders, and providing general health information. By handling these responsibilities, chatbots alleviate the load on healthcare systems, allowing medical professionals to focus more on complex care tasks.

The rapid growth and adoption of AI chatbots in the healthcare sector is exemplified by ChatGPT. Within a mere five days of its launch, ChatGPT amassed an impressive one million users, and its user base expanded to 100 million users in just two months [4]. A study conducted six months ago on the use of AI chatbots among healthcare workers found that nearly 20 percent of them utilized ChatGPT [5]. This percentage could be even higher now, given the increasing reliance on AI chatbots in healthcare.

The swift adoption of ChatGPT and similar technologies highlights the growing importance and impact of AI chatbots in transforming healthcare services and enhancing patient care. As AI chatbots continue to evolve and improve, they are expected to play an even more significant role in healthcare, further streamlining processes and optimizing resource allocation.

Limitations of AI chatbots

While AI chatbots offer many benefits, it is critical to understand their limitations. Currently, AI lacks the capacity to demonstrate empathy, intuition, and the years of experience that medical professionals bring to the table [6]. These human traits are invaluable in effective patient care, especially when nuanced language interpretation and non-verbal cues come into play. AI chatbots are limited to operating on pre-set data and algorithms; the quality of their recommendations is only as good as the data fed into them, and any substandard or biased data could result in harmful outputs.

The irreplaceable role of medical professionals

The role of a medical professional is far more multifaceted than simply diagnosing illnesses or recommending treatments. Physicians and nurses provide comfort, reassurance, and empathy during what can be stressful and vulnerable times for patients [6]. This doctor-patient relationship, built on trust, rapport, and understanding, is not something that can be automated or substituted with AI chatbots. Additionally, while chatbots can provide general health information and manage routine tasks, their current capabilities do not extend to answering complex medical queries. These queries often require deep medical knowledge, critical thinking, and years of clinical experience that chatbots do not possess at this point in time [7]. Thus, the intricate medical questions and the nuanced patient interactions underscore the indispensable role of medical professionals in healthcare.

Examples of AI chatbots use in healthcare limitations and capabilities

AI chatbots have been increasingly integrated into the healthcare system to streamline processes and improve patient care. While they can perform several tasks, there are limitations to their abilities, and they cannot replace human medical professionals in complex scenarios. Here, we discuss specific examples of tasks that AI chatbots can undertake and scenarios where human medical professionals are still required.

AI Chatbot Capabilities

Appointment Scheduling: AI chatbots can manage appointments, allowing patients to easily book, reschedule, or cancel appointments without human intervention.

Prescription Refill Reminders: Chatbots can send timely reminders to patients to refill their prescriptions, enhancing medication adherence.

Symptom Checker: AI chatbots can analyze the symptoms reported by patients and provide possible diagnoses based on the input.

Patient Triage: Chatbots can classify patients based on the severity of their condition, helping healthcare providers prioritize cases and allocate resources efficiently.

Mental Health Support: AI chatbots can offer psychological support, providing users with coping strategies and resources for mental health issues.

Medical Research Assistance: AI chatbots can support medical research by helping researchers find relevant literature, analyze data, and generate summaries or reports. They can also aid in writing research papers, grant proposals, or reviews by providing suggestions, identifying gaps in knowledge, and offering language assistance [5].

Complex scenarios requiring human medical professionals

Diagnosis of Rare or Complex Conditions

While AI chatbots can provide preliminary diagnoses based on symptoms, rare or complex conditions often require a deep understanding of the patient's medical history and a comprehensive assessment by a medical professional.

Surgical Procedures

AI chatbots cannot perform surgeries or invasive procedures, which require the expertise, skill, and precision of human surgeons.

Counseling and Empathy

Although AI chatbots can provide support and resources for mental health issues, they cannot replicate the empathy and nuanced understanding that human therapists offer during counseling sessions [6,8].

Interpretation of Complex Diagnostic Tests

Some diagnostic tests, such as MRIs, CT scans, and biopsy results, require specialized knowledge and expertise to interpret accurately. Human medical professionals are better equipped to analyze these tests and deliver accurate diagnoses.

Treatment Plan Development

While AI chatbots can provide general recommendations, developing personalized treatment plans based on a patient's unique circumstances, medical history, and preferences often requires the judgment and expertise of human healthcare providers.

Ethical and legal considerations

Integrating AI into healthcare presents various ethical and legal challenges, including questions of accountability in cases of AI decision-making errors. These issues necessitate not only technological advancements but also robust regulatory measures to ensure responsible AI usage [3]. The increasing use of AI chatbots in healthcare highlights ethical considerations, particularly concerning privacy, security, and transparency. To protect sensitive patient information from breaches, developers must implement robust security protocols, such as encryption. Ethical considerations extend to ensuring transparency in chatbot interactions, obtaining proper consent for data collection and use, and establishing clear guidelines for chatbot use in clinical settings to prevent misuse or misinterpretation. Addressing these ethical and legal concerns is crucial for the responsible and effective implementation of AI chatbots in healthcare, ultimately enhancing healthcare delivery while safeguarding patient interests [9].

Future prospects- collaboration over replacement

AI chatbots are undoubtedly valuable tools in the medical field, enhancing efficiency and augmenting healthcare professionals' capabilities. They could be particularly beneficial in areas with limited healthcare access, offering patient education and disease management support. However, considering chatbots as a complete replacement for medical professionals is a myopic view. The more plausible and beneficial future lies in a symbiotic relationship where AI chatbots and medical professionals complement each other. Each, playing to their strengths, could create an integrated approach to healthcare, marrying the best of digital efficiency and human empathy. As we journey into the future of medicine, the narrative should emphasize collaboration over replacement. The goal should be to leverage both AI and human expertise to optimize patient outcomes, orchestrating a harmonious symphony of humans and technology.

While advancements in AI and machine learning could lead to more sophisticated chatbots, their potential to entirely replace medical professionals remains remote. The integration of AI chatbots and medical professionals is more likely to evolve into a collaborative approach, where professionals focus on complex medical decision-making and empathetic patient care while chatbots supplement these efforts. This future, however, depends on various factors, including technological breakthroughs, patient and provider acceptance, ethical and legal resolutions, and regulatory frameworks.

In conclusion, it is paramount that we remain steadfast in our ultimate goal of improving patient outcomes and quality of care in this digital frontier. By restating the main themes discussed around the complementary role of AI chatbots and the need for collaboration between AI and medical professionals, we emphasize that AI chatbots represent a powerful supplement to healthcare, but they should not be seen as a complete substitute for the irreplaceable role of medical professionals.
